# A Method for Evaluation the Fatigue Microcrack Propagation in Human Cortical Bone Using Differential X-ray Computed Tomography

**DOI:** 10.3390/ma14061370

**Published:** 2021-03-11

**Authors:** Petr Koudelka, Daniel Kytyr, Tomas Fila, Jan Sleichrt, Vaclav Rada, Petr Zlamal, Pavel Benes, Vendula Bendova, Ivana Kumpova, Michal Vopalensky

**Affiliations:** Institute of Theoretical and Applied Mechanics, Czech Academy of Sciences, Prosecka 809/76, 19000 Praha 9, Czech Republic; kytyr@itam.cas.cz (D.K.); fila@itam.cas.cz (T.F.); sleichrt@itam.cas.cz (J.S.); rada@itam.cas.cz (V.R.); zlamal@itam.cas.cz (P.Z.); benes@itam.cas.cz (P.B.); bendova@itam.cas.cz (V.B.); kumpova@itam.cas.cz (I.K.); vopalensky@itam.cas.cz (M.V.)

**Keywords:** human cortical bone, low-cycle fatigue, microcracks, digital volume correlation, computed tomography

## Abstract

Fatigue initiation and the propagation of microcracks in a cortical bone is an initial phase of damage development that may ultimately lead to the formation of macroscopic fractures and failure of the bone. In this work, a time-resolved high-resolution X-ray micro-computed tomography (CT) was performed to investigate the system of microcracks in a bone sample loaded by a simulated gait cycle. A low-cycle (1000 cycles) fatigue loading in compression with a 900 N peak amplitude and a 0.4 Hz frequency simulating the slow walk for the initialization of the internal damage of the bone was used. An in-house developed laboratory X-ray micro-CT imaging system coupled with a compact loading device were employed for the in situ uni-axial fatigue experiments reaching a 2μm effective voxel size. To reach a comparable quality of the reconstructed 3D images with the SEM microscopy, projection-level corrections and focal spot drift correction were performed prior to the digital volume correlation and evaluation using differential tomography for the identification of the individual microcracks in the microstructure. The microcracks in the intact bone, the crack formation after loading, and the changes in the topology of the microcracks were identified on a volumetric basis in the microstructure of the bone.

## 1. Introduction

As a part of the research devoted to the damage and remodeling of a human cortical bone, the processes and mechanisms related to the influence of imperfections and the fracture characteristics have been intensively studied [1]. It has been assessed that the two major types of damage at the microscale are linear microcracks, with a length in the range of 50–100 μm, clusters of sublamellar sized cracks, forming diffuse damage [2], are the two major types of damage at the microscale. In a healthy bone, the matrix repair stimulated by the microstructural interface enables the effective remodeling of the bone such that the micrometric damage remains clinically unnoticed. From a mechanical standpoint, an engineering analogue of a cortical bone is a fibre-reinforced composite, where, during the fatigue loading, both phases allow the formation of matrix damage and energy dissipation at the microstructural interfaces that limit the microcrack propagation. In the bone itself, energy dissipation occurs at the mineral and collagen interfaces, lamellae, and tissue heterogeneity among the osteons [3]. As a result of aging or disease-induced remodeling, a sustained loading at levels excessive for damage tolerance of the bone causes unlimited microcrack propagation, which may result in a microdamage accumulation together with a loss of tissue heterogeneity and a reduction of its macroscopic fracture toughness [4,5].

The understanding of the underlying processes is important also from a material characterization standpoint to enable the proper analytical and numerical modeling of the bone. In this regard, one of the necessary steps is the assessment of the fatigue-induced formation of microcracks influencing the effective mechanical properties of the bone and its load-bearing capability. Fragility fractures have been studied on the basis of epifluorescence microscopy with 3D reconstruction for the visualization of the microcracks together with the geometrical characterization [6]. It has been demonstrated that the optical observation of the defects on the microscale has a potential to become a tool in the calculation of the stress intensity for indicating the probability of an individual propagating microcrack to cause a stress or fragility induced fracture. However, the use of optical microscopy is limited in the ability to study microcracks only in a limited volume of interest. To fully understand the influence of fatigue on the bone fragility, it is necessary to utilize nondestructive testing methods with the ability to inspect the three-dimensional volume of interest, which inevitably leads to the utilization of radiographical methods and particularly X-ray imaging. Even though transmission radiography enables as high as nanoscale resolution in the case of synchrotron radiation sources [7], computed tomography (CT) is a preferred method suitable for the visualization of three-dimensional microcracks in a compact bone [6]. Here, X-ray micro-CT (XCT) imaging allows the time-lapse observation of the damage initiation and propagation [8], also using imaging devices based on laboratory X-ray systems [9], where a single-micron resolution has been routinely achieved on the recent setups [10,11,12]. Furthermore, laboratory X-ray CT scanners are effectively used in numerous fields. Additive manufacturing is a typical example because the X-ray based geometric analysis coupled with the imperfection detection helps to optimize the manufacturing process and increase the load bearing capacity and durability of the parts [13,14]. Other applications can be found in, e.g., corrosion inspection and crack propagation in civil engineering materials [15,16].

To achieve the best possible quality of the CT reconstruction suitable for the investigation of the linear microcracks even without synchrotron radiation, it is necessary to use an enhanced methodology of the time-lapse XCT imaging and post-processing protocol. This comprises the in situ fatigue loading of the sample, X-ray source focal spot movement correction, and the post-processing of the dataset using a differential tomography approach coupled with a digital volume correlation (DVC) as a basis for the volume registration procedure [17,18,19]. DVC is an extension of the digital image correlation procedure to three dimensions, which can be effectively used for two different purposes. Firstly, it is possible to use DVC to obtain the parameters of the rigid transformation that can be used to shift a volume in the 3D space to align it with another volume. The resulting registered volumes are then suitable for differential tomography procedures to highlight and emphasize microstructural changes in the material as a result of the mechanical loading, provided that the precision of the registration is sufficiently high. Typically, such a calculation is performed using a reference volume in terms of the intact sample and the volumes of the sample subjected to a loading procedure. Secondly, the DVC, using a higher order transform, such as the full-affine transform, is a method for the assessment of the full-field displacement and strain fields of an investigated object subjected to intermittent or continuous loading with the ability to identify, e.g., the strain localization and stress concentration that are both important aspects of bone mechanics. Although the DVC method has the potential to reveal subpixel, i.e., sub-micron, deformations, its accuracy and precision can be affected by many factors including the imaging instrumentation (X-ray source stability, movement precision of the positioning stages, etc.), the sample geometry and its inner structure (material, porosity, etc.), the tomographic reconstruction algorithms, and the reconstructed 3D image post-processing (artifact reduction, resulting contrast-to-noise ratio, etc.). All of these factors are important and have to be taken into account during the DVC evaluation procedure.

In this paper, we used a time-resolved high-resolution XCT to investigate the formation of linear microcracks in a human cortical bone induced by uni-axial in situ low-cycle compressive fatigue loading. We used an approach similar to the study of Fernández et al. [9], however, as a part of research concentrated on the mechanical characteristics of the bone and bone tissue engineering, the aim of the work was to explore the capability of a local DVC approach to assess the correlation between the regions of damage accumulation with the displacement concentration calculated using the DVC. A laboratory X-ray imaging instrumentation was used deliberately despite its inherent resolution limits to investigate its suitability in contrast to the work of other authors, where the synchrotron radiation was used to achieve a sub-micron resolution in the reconstructed 3D images (e.g., [7,8]). The samples were loaded by an in-house designed device integrated into a modular laboratory micro-CT scanner. A CT scan of the sample in the intact state was performed to obtain the reference state for the subsequent deformation analysis. The loading procedure, based on a force-controlled cyclic loading, was based on multiple loading increments composed of several thousand loading cycles. Between the individual loading increments, a tomographical scan of the samples was performed. The resulting sets of reconstructed volumes were processed using a custom DVC procedure and used for the qualitative evaluation of the crack propagation using differential tomography. We show that the formation of a system of microcracks can be studied using instrumentation based on a standard high-resolution X-ray scanner with a micrometric resolution, when the proposed methodology comprising in situ loading is used.

## 2. Material and Methods

### 2.1. Specimens

For the analysis, a human proximal femur (male donor, 52 years) stored in a formaldehyde solution over a long-term period was used as a source of the cadaveric cortical bone tissue. Although long-term preservation has a measurable influence on the material properties of the bone, represented typically by a 5–15% decrease in the Young’s modulus and yield stress as reported during quasi-static tests [20,21], such a difference in the mechanical properties has a negligible influence regarding the aim of the presented research. An oscillating saw was used to extract a bone segment with a height of 15mm from the area inferior to the trochanter minor femoris. The motivation behind the selection of this anatomic location was the peak loading concentration during physiological activities and because it frequently suffers from sub-trochanteric fractures [22]. Then, drilling took place in the inferior direction (in parallel to the femoral shaft) using a combination of a fine laboratory drill and a diamond-coated hollow drill bit was used to obtain cylindrical bone samples (diameter 2.8–2.9 mm). A drilling speed of $2\;\mathrm{mm}\cdot \min^{-1}$ at 800rpm was used and the thermal effects of the drilling were reduced by immersion in a cooling lubricant. As a result, the drill bit temperature measured directly after drilling did not exceed the temperature of $40{\;}^{\circ }C$ and the negative effects of the sample preparation were minimized [23]. The final samples with a length of 5–9 mm were finished by grinding on their top and bottom faces to ensure a proper shape for the mechanical testing with plane-parallel faces including the removal of the volume influenced by cutting.

### 2.2. Mechanical Loading

**Figure 1:**
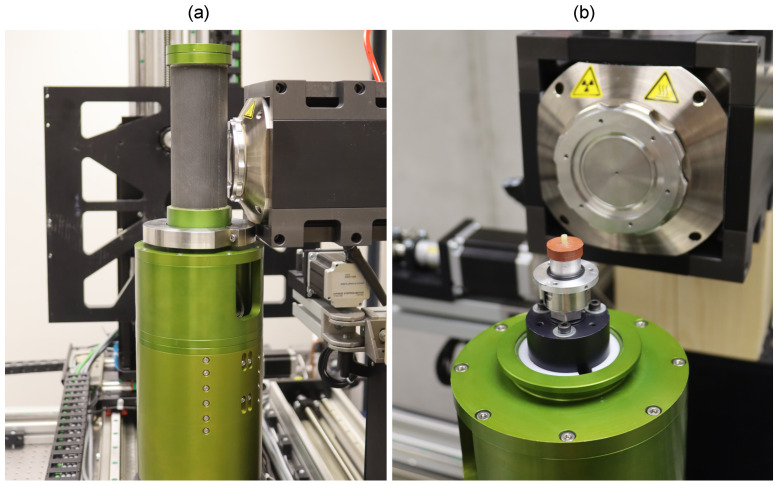
The experimental setup with the in situ loading device mounted in the X-ray scanner: (**a**) the scanning position for maximal magnification and (**b**) a detailed view on the open device with the sample placed for testing.

All the mechanical experiments were performed using an in-house developed table top loading device, in detail originally presented in [24], allowing for the in situ 4D XCT biomechanical experiments with modifications for low-cycle fatigue loading and high resolution imaging [25]. The specimen was placed in the in situ loading device in a hollow circular plate. The device itself was directly mounted on the rotary stage of the X-ray scanner (see Figure 1).

**Figure 2:**
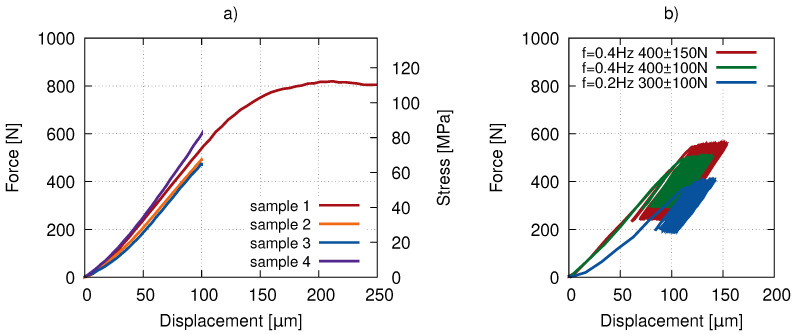
The loading curves of the preliminary compression tests: (**a**) quasi-static loading in an elastic and post-yield regime and (**b**) the low-cycle fatigue response.

Before the fatigue experiment involving the X-ray imaging, a batch of pilot experiments was carried out using the same uni-axial loading device. Displacement-driven quasi-static loading was performed with a loading rate of $1.0\;\mu m\cdot s^{-1}$ and $0.5\;\mu m\cdot s^{-1}$ for Samples 1 and 2 (Figure 2a red hues) and Samples 3 and 4 (Figure 2a blue hues), respectively. The results of the quasi-static tests are presented in Figure 2a) showing the elastic modulus in the range of 4–6 GPa and a plateau stress of approximately 110 MPa for Sample 1. Low-cycle testing up to 1000 cycles was performed (see Figure 2b) with different mean force values and amplitudes to evaluate the displacement during the loading.

Based on the results of the quasi-static test and the low-cycle testing in the elastic region, the parameters for the final test were identified. The in situ loading procedure was composed of the initial loading beyond the elastic limit of the specimen to induce the initial defects into the specimen’s microstructure. Here, the first tomographical scan before the first cyclic loading served as a reference for the evaluation of the fatigue cracks using the differential tomography. On the specimen, a miniature lead marker (diameter <0.5mm) was placed near its transversal plane to serve as a defined reference point to identify the X-ray tube focal spot movement during the tomography [26].

**Figure 3:**
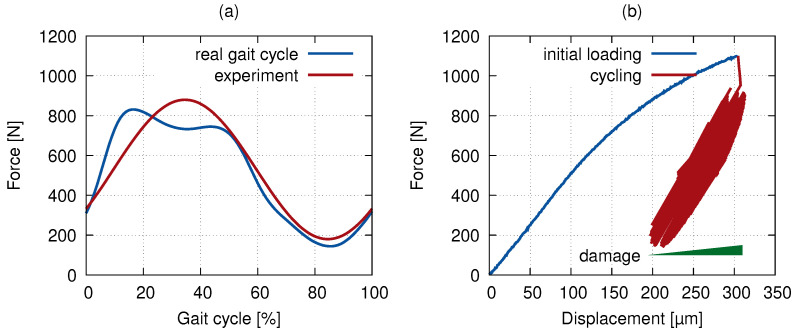
In situ loading: (**a**) the loading function used in the experiment and a real gait cycle and (**b**) the force–displacement curve of the actual loading in the computed tomography (CT) scanner.

The force-driven tests with a sine loading function were carried out at a frequency of 0.4 Hz to simulate the slow walk gait cycle [27,28] (see Figure 3a). The resulting curves of the 1000 gait cycles are plotted in Figure 3b. The horizontal shift in the loading curve implicates the plastic deformation coupled with the development of the bone micro-damage described in Section 3.

In contrast to the time-lapse tomographical measurements under loading performed for the structural characterization of porous solids at a mesoscopic level, the in situ radiographical imaging in this work was performed in an unloaded state for two reasons. Firstly, due to the width of the sought microcracks at double of the effective voxel size achieved in the experiments, it was necessary to pursue the highest possible quality of the reconstructed 3D images. Here, the CT scan performed under loading (at, e.g., the mean load level) would be affected by the material relaxation effects resulting in a blur in the reconstructed 3D image even after a settling time of over an hour. Secondly, the compressive stress distribution in the microstructure of the bone led to the closing of the micro cracks, which effectively precluded the experimental study.

### 2.3. Radiographical Imaging and Computed Tomography

The 4D micro-CT measurements were performed using a TORATOM modular X-ray imaging device capable of performing dual-source/dual-energy measurements, and which can be equipped with different types of X-ray sources and detectors. For the purpose of this study, we used an XWT-160-THCR (X-RAY WorX, Hanover, Germany) [29] transmission type X-ray tube with a maximum tube voltage of 160kV, a maximum target power of 25W and a minimum focal spot size of 1μm in a nanofocus emission mode. A dexela 1512 NDT (PerkinElmer, Waltham, MA, USA) [30] CMOS flat panel detector equipped with a high-energy GOS scintillator was used for the acquisition of the radiograms. The effective X-ray energy range of the detector is 12–225 keV, making it suitable to capture of the bone samples. The native resolution of the pixel matrix is 1944×1536px with a pixel pitch of 74.8μm yielding an active area of 145.4×114.9mm. The maximum achievable frame rate, in the case of non-binned acquisitions, is 26 fps.

To reach the highest possible geometrical magnification, the loading device with the specimen was placed as close as possible to the focal spot of the X-ray source resulting in approximately a 33 mm focus–object distance thanks to the use of a low diameter carbon fibre tube. The focus–detector distance was set to 1233 mm resulting in a 37.2× geometrical magnification in the projections and an effective pixel size of approximately 2μm. The tube was operated at an acceleration voltage of 80 kV, a target current of 28μA, and a target power of 2.2 W in a nanofocus mode to reach the lowest possible spot size of 1μm. The acceleration voltage was selected with respect to the attenuation characteristics of the bone tissue to reach the optimum contrast in the radiograms. We have already shown in [31] that in a simplistic model of a monochromatic source, the optimum voltage can be chosen based on the intensity ratios in the radiographic images. In spite of its simplicity and neglecting the polychromatic nature of the X-ray tube beam, the experiments confirmed such an approach to be plausible. Such a procedure was, therefore, used to optimize the tube voltage for the experiments in this work. During every tomographical scan, 1800 angular projections were acquired by averaging three exposed images at each angular position and the detector was set to a 2 s acquisition time in a higher-sensitivity low-well mode. A projection-level correction was performed using tools from the detector’s API using correction data derived from a median of 100 exposures in the open-beam and dark-field images.

**Figure 4:**
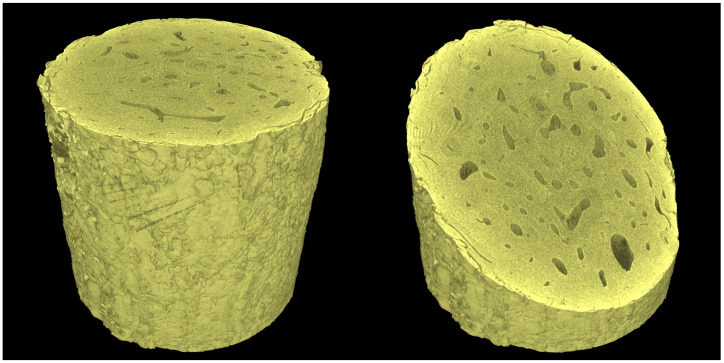
Visualization of the investigated sample based on the reconstructed 3D image in the intact state.

It is a well-known fact that during long-term tomographical scans various error sources may lead to a reduced quality in the imaging yielding various artefacts in the reconstructed 3D images or blurred data. We have analyzed this phenomenon in our previous studies, where we have also demonstrated various compensation methods [26]. In this work, we used spot-movement corrected imaging due to the length of the individual tomographical scans (approximately 75 min) and the setting of the X-ray tube, where a maximum target current causes significant thermo-mechanical drifts and impermanence in the tube due to the high stabilization current for focusing the electron beam. To compensate for these effects, every tomographical scan was divided into 10 individual sub-scans composed of 180 angular projections dispersed equiangularly over one full rotation. These individual sectional measurements were mutually shifted by 0.4 degrees yielding a total of 1800 equiangular projections for the entire tomographical scan. During every CT sub-scan, a reference image in an identical angle of rotation was captured and the resulting series of images was used to calculate the image offsets in the CT scan due to the X-ray tube focal spot drift. The offsets were calculated using the digital image correlation (DIC) procedure applied on the individual reference projections. Every projection in the sub-scan was then corrected by shifting the projections in the direction opposite to the calculated focal spot drift by a displacement established from the shifts between the first and the last reference image in the sub-scan by the linear interpolation. As a result, a significant sharpening of the reconstructed 3D volume was achieved. The volumes were reconstructed using a filtered back-projection (FBP) algorithm in a CT reconstruction module embedded in VG Studio Max 3.4 (Volume Graphics, Heidelberg, Germany) software. The software uses an extended convolution-backprojection formula for the direct reconstruction of a three-dimensional density function from a set of two dimensional projections based on a method introduced by Feldkamp, Davis and Kress (FDK algorithm) [32]. This algorithm has become a solution implemented in numerous commercially available CT imaging systems and reconstruction tools in either its original form or in various modifications [33]. Further details regarding the FBP method can be found in, e.g., [34] or [35]. To avoid the presence of ring artifacts, the data have to be calibrated on multiple levels starting at the projection level, where the detector’s defective pixels are corrected using a combination of flat field correction, bad pixel correction, and speckle removal. Here, the flat field correction is performed using the open-beam and dark-field images processed using tools embedded in the detector’s API, the bad pixel correction is carried out by interpolation based on a mask of known bad pixels, and the speckle removal, which is performed by the reconstruction software, is based on the interpolation of the remaining pixels present in all the projections with an excessive difference in the intensity compared to their neighborhood. The reconstruction software also offers the tool to perform a ring artifact reduction on the resulting tomographic slices of the reconstructed 3D image, where the concentric rings of the ring artifact are reduced by interpolation with respect to a given neighborhood. However, neither this tool nor the external treatment of the data, e.g., the median filtering of the slices in polar coordinates, was used in this work due to the fact that the filtering leads to a loss of information that may prevent the identification of the thin microcracks in the bone. A demonstration of the reconstructed volume is shown in Figure 4.

Because the subtraction of the loaded-state volume from the reference one is insufficient for the identification of the microcracks, the so-called blending procedure was used for the differential tomography. The blending of the volumes consists of an arbitrary combination of operations performed on the reference and loaded-state volume, including addition, subtraction, and multiplication. In this work, the volume of the loaded state was multiplied by an integer and then summed with the reference volume. Hence, the resulting blended volume $V_{b}$ is calculated using the reference volume $V_{r}$ and the loaded-state volume $V_{l}$ according to $V_{b}=V_{r}+mV_{l},\;m\in Z$. The value of the multiplier *m* was obtained in an iterative procedure, where its value varied in the interval $m\in \langle 2;50\rangle$ and the resulting blended volume was inspected for contrast between the void space and the bone tissue. In this work, the value m=20 was identified as an optimum for the precise measurement of the microcrack geometry.

**Figure 5:**
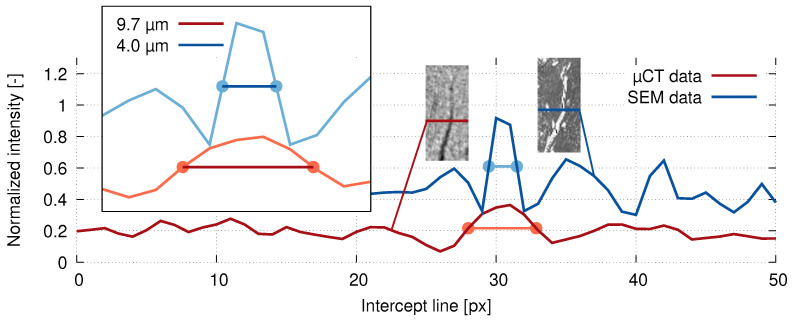
The crack thickness calculation based on the full-width half-maximum (FWHM) method using the line intensity profiles based on the SEM (blue) and XCT (red) data.

However, even though the resolution of the reconstructed volumes was 2μm allowing for the microcrack evaluation, it was insufficient for a single osteon level. For this reason, an SEM inspection was carried out on the same bone sample as a comparative imaging method. Due to the need of embedding the sample for the preparation prior to the SEM imaging, the sample after the in situ loading procedure was used. The sample of the bone was prepared for scanning by fixation in a low viscosity resin and a layer with a thickness of 700μm (±40μm on a diameter of 25 mm) was ground out and then polished using 0.25μm aluminum oxides. Using SEM (Mira II, Tescan, Brno, Czech Republic) [36], a secondary electron emission scan of the whole cross-section of the bone with a pixel size of 1μm was acquired and used as a comparative image for the full-width half-maximum (FWHM) analysis of the tomographic imaging quality. Using the known layer thickness of the material removed by grinding and by comparison of distinctive microstructural features, the respective slices in the reconstructed 3D images were identified. In both the SEM image and the CT slice from the reconstructed 3D image of the loaded sample, a representative crack was used as a basis for the FWHM calculation. Figure 5 shows the calculated FWHM for both image sources superimposed over a plot of relative intensity versus the spatial coordinates together with the visualization of the crack used for the analysis and the corresponding intercept lines, where the intensity data were acquired. It can be seen that the FWHM of the CT scan at 9.7μm was 2.425× higher than the FWHM of the SEM image at 4μm.

### 2.4. Digital Volume Correlation

Because the differential tomography is based on identifying the changes in the analyzed volumes by blending the two volumes, the precise alignment of the volumes is a fundamental requirement to guarantee its precision and reliability. Here, even a shift in a few voxels may either prevent the analysis of the micro-CT data or significantly reduce resolution in the differential volume. As can be seen in the representative force-displacement of the experiments (see Figure 3b), the cyclic fatigue loading of the specimens causes the permanent deformation in terms of a height reduction by several tens of microns and, thus, tens of voxels. For this reason, this shift has to be evaluated prior to the employment of the differential tomography procedure. Since this task is equivalent to the evaluation of the displacement and strain fields in a standard in situ time-resolved CT, the DVC algorithm was applied to obtain the transformation parameters necessary for the precise alignment of the volumes during the differential tomography.

The DVC method is an extension of the DIC into three spatial dimensions. During the DVC procedure, the virtual correlation sub-volumes are defined in the volume of interest (VOI) and tracked throughout the sequence of the tomographical scans (in the case of the standard in situ time-resolved CT) or between pairs of tomographical scans, where the reference VOI is always the CT in an unloaded state (in the case of the differential tomography). The tracked sub-volume is defined as a cube-shaped domain formed around the centroid of the respective correlation sub-volume. The reference cube-shaped sub-volume deforms between the individual loading states as a result of the specimen’s inner structure deformation, while the DVC algorithm establishes its location in the 3D image of the deformed state from the extremum of the correlation coefficient calculated using the selected criterion.

In this paper, the DVC procedure was used in two different forms depending on the purpose of the obtained result. First, to be able to inspect, measure, and visualize the system of microcracks within the bone, a local alignment of the volumes was performed. Here, the morphological differences between the reference and loaded state were highlighted using the blending procedure applied to the uncorrelated volumes and the volumes of interest were defined by a manual inspection of the blended volume. Then, the reference and loaded volume were aligned locally for each volume of interest containing the cracks by a correlation of 125 voxels close to the end of the crack, but far enough from the crack to prevent any influence of the void on the correlation process. On the basis of the correlation procedure, the points with sufficiently high correlation coefficient were used to calculate a general transformation matrix to transform the loaded volume for the best achievable alignment with the reference volume. This procedure was performed using the least square method with the tools implemented in the MATLAB global optimization toolbox.

**Figure 6:**
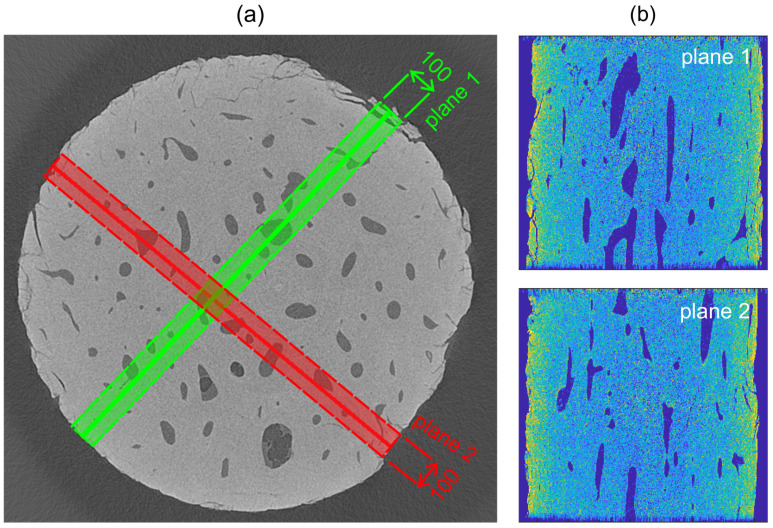
The selected planes for the digital volume correlation (DVC) procedure: (**a**) visualization of the sub-volume with a thickness of 100 voxels and the selected sub-volume medial planes (shown by a line) for the DVC procedure and (**b**) visualization of the plane 1 (top) and plane 2 (bottom) of the unloaded sample.

After that, to obtain the displacement and strain fields in the regions of interest, where the stress concentration was anticipated, the DVC procedure was performed on two perpendicular planes (see Figure 6). As an input for the DVC algorithm, a sub-volume with a thickness of 100 voxels was taken for each plane and the correlation procedure was performed on its medial sub-plane (i.e., its plane of symmetry). Our in-house DVC algorithm tracked the deforming sub-volumes using a two-step procedure to assess the correlation coefficients initially at the voxel level and consequently at the sub-voxel level to determine the 3D displacement vectors more accurately [37].

At the voxel level, a combination of the steepest-gradient and normalized cross-correlation method (NCC) is used to calculate the integer value of the voxel displacement. Then, an estimation at the sub-pixel level is performed as the integer values of the displacement vector are passed on as the initial values to a combination of interpolation and a modified Lucas–Kanade tracking algorithm [38] extended to 3D [39,40,41], which accounts for the deformations of the sub-volume.

The initial position of each node (where the node stands for every vertex of the eight-node voxel) in the analyzed sub-volume was established in the reference volumetric model representing the unloaded specimen. The changes of node positions in the sub-volume were calculated using the reconstructed 3D image representing the loaded state of the specimen. Nodal displacements in every load-step were evaluated by a subtraction of the actual nodal coordinates in the deformed volume from the reference (initial) positions. The affine transformation matrix of every voxel was then calculated from the known displacement values for the nodes constituting the voxel to determine the Green–Lagrange strain tensor from the affine transformation matrix and deformation gradient tensor.

In this work, the noise present in the reconstructed volume prevented the application of the full-field DVC procedure directly and the volumes filtered using the 3D median filter with the kernel size of 13 voxels before the DVC procedure was commenced. The location of every voxel in the reconstructed volume was used as a centroid of 18×18×18 voxel sub-volume (correlation window) containing $5.832\times 10^{3}$ voxels in total used in the correlation procedure, while the offset (i.e., a distance from the centroid of the sub-volume, where the correlation is evaluated) was set to 24 voxels. The size of the correlation window was determined based on an optimal correlation window size analysis (see Section 2.4.1).

Due to computational costs of the DVC procedure, only the two planes containing approximately $2\times 10^{6}$ voxels and $4\times 10^{6}$ nodes perpendicular to each other (see Figure 6b) were selected to assess the geometrical changes in the specimen as a result of the fatigue loading. Voxels of each plane were divided to eight subsets equally sized in terms of the number of voxels for parallelization of the DVC calculation. The DVC evaluation within every subset took approximately 2.42 s per one node resulting in a 346 h-long calculation time. A high-performance workstation, based on a Xeon E5-2660 v4 (Intel, Santa Clara, CA, USA) CPU, was used and three threads were dedicated for each subset. The final displacement field was obtained using a two-step filtering procedure to correct the invalid displacement values (where an unconverged solution for a given node occurred) and the non-physical values of the displacement (erroneous values of the displacement not satisfying the principle of continuity and smoothness of the local deformation gradients).

#### 2.4.1. Analysis of Optimal Correlation Window Size

**Figure 7:**
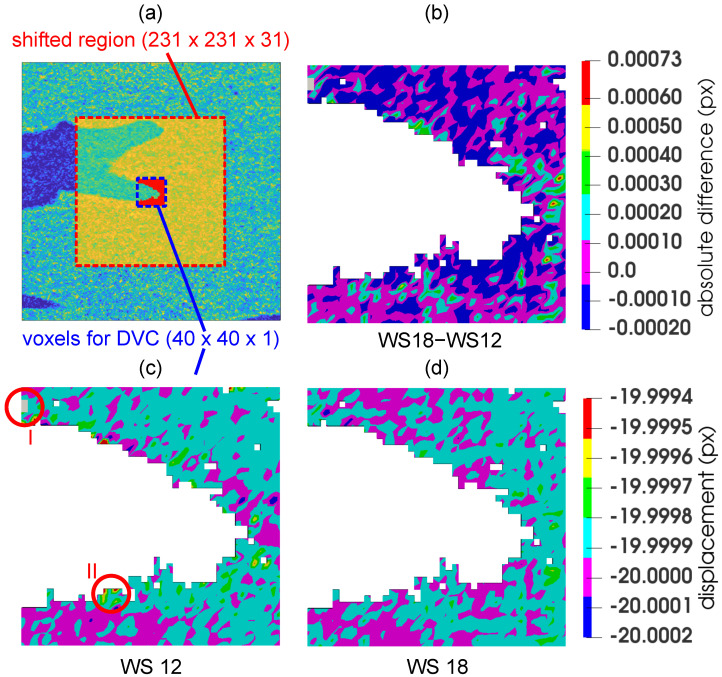
Determination the of optimal correlation window size: (**a**) red box-the dataset subjected to the in-plane numerical rigid body motion, blue box-the region, where the displacement was evaluated using the DVC, (**c**,**d**) the vertical displacement fields calculated using a correlation window size of 12 px and 18 px, and (**b**) the difference field calculated using the results of the two correlation windows. The circular regions “I” and “II” depict the unconverged solution in grey and highest error in this sub-volume, respectively.

To establish an optimal correlation window dimensions with respect to both the precision and computational costs of the DVC procedure, a virtual (i.e., numerical) experiment was performed with the reconstructed 3D image of the unloaded specimen. From its centroid, a cube with the dimensions of 231×231×31 was selected and subjected to an in-plane numerical rigid body motion in two directions, which was prescribed in five steps of 4 voxels (8.041μm) yielding a total displacement of 20 voxels (40.206μm) in each direction. In the non-shifted state, the non-void voxels (formed from 2214 nodes) belonging to the region with dimensions of 40×40×1 were chosen for the DVC calculation (see Figure 7a).

The DVC procedure was then applied to the numerically displaced data and the calculated displacement field was used for the assessment of the sensitivity of the DVC algorithm to the size of the correlation window and to estimate the systematic errors of the DVC procedure. The optimum size of the correlation window was determined in a parametric study comprising the correlation window set in the range of 6 to 20 voxels (even values only). Here, the tracking quality of the algorithm was analyzed using standard deviation of the difference between the synthetic (numerically prescribed) displacements and the displacement calculated by the DVC algorithm evaluated from the convergence through all the virtual load steps. Comparing the known numerical displacements with the calculated displacements, it was possible to evaluate the mean-bias error (MBE) and root-mean square error (RMSE) at the last virtual load step according to [42]:

(1) $$\mathrm{MBE}=N^{-1}\sum_{i=1}^{N}\left(P_{i}-O_{i}\right),$$

(2) $$\mathrm{RMSE}={\left[N^{-1}\sum_{i=1}^{N}{\left(P_{i}-O_{i}\right)}^{2}\right]}^{\frac{1}{2}},$$

where *P* is the expected value (the known prescribed numerical displacement), *O* is the displacement calculated using the DVC algorithm, and *N* is the total number of the correlation points. Thus, MBE (or bias) represents the overall bias error, whereas RMSE is a measure of average deviation from the calculated value (in contrast to the standard deviation, which is related to deviations from the mean) emphasizing the largest errors in the evaluated set.

**Figure 8:**
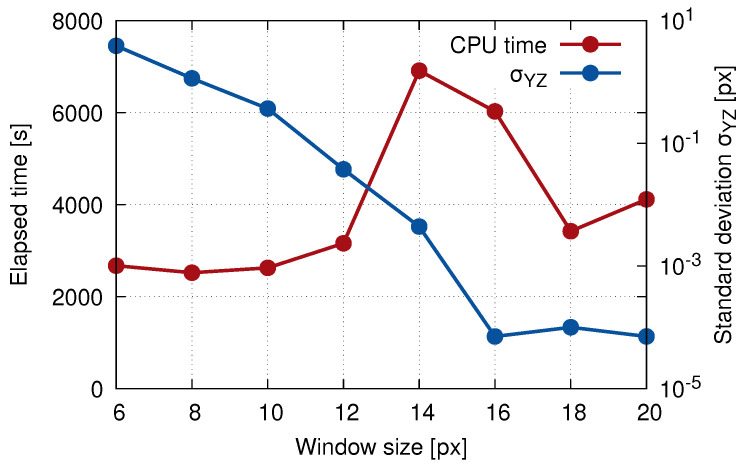
The elapsed time of the calculation and standard deviation plotted against the correlation window size showing the result of the DVC precision evaluation.

Based on the results of the parametric study (see Table 1) and taking into account also the computational time (e.g., the correlation window size of 16 had a lower error, but its computational time was doubled), the resulting correlation window size of 18 was chosen for the DVC calculation of the displacement field of the selected plane 1 and 2 (see the corresponding graphs in Figure 8). It can be noticed that the computation time increased up to the 14 pixel window size and then rapidly decreased to a local minimum for 18 pixels. This effect was caused by the implementation of functions in the MATLAB toolkits embedded in our DVC algorithm that offered user-independent scalability by invoking parallel operations, when given criteria were met. For example, the execution of the DVC algorithm with the window size 18 of pixels used approximately 1.6 times more processor power (i.e., CPU threads) on our HPC workstation in comparison to the windows size of 14 pixels (with the same user-defined settings).

Figure 7 depicts a vertical displacement field calculated using the correlation window with a size of 12 pixels (Figure 7c), 18 (Figure 7d) and a difference field (Figure 7b). Although the computational time of these window sizes was similar, the error was significantly higher in the case of the window size of 12px. The circular region denoted “I” shows in grey an unconverged solution, while the circular region “II” indicates one of the regions with the highest error. The difference field between the window size of 12px and 18px highlights where the values of the displacement of these two DVC procedures were almost identical (zero value) and where, on the contrary, they differed significantly.

## 3. Results

**Figure 9:**
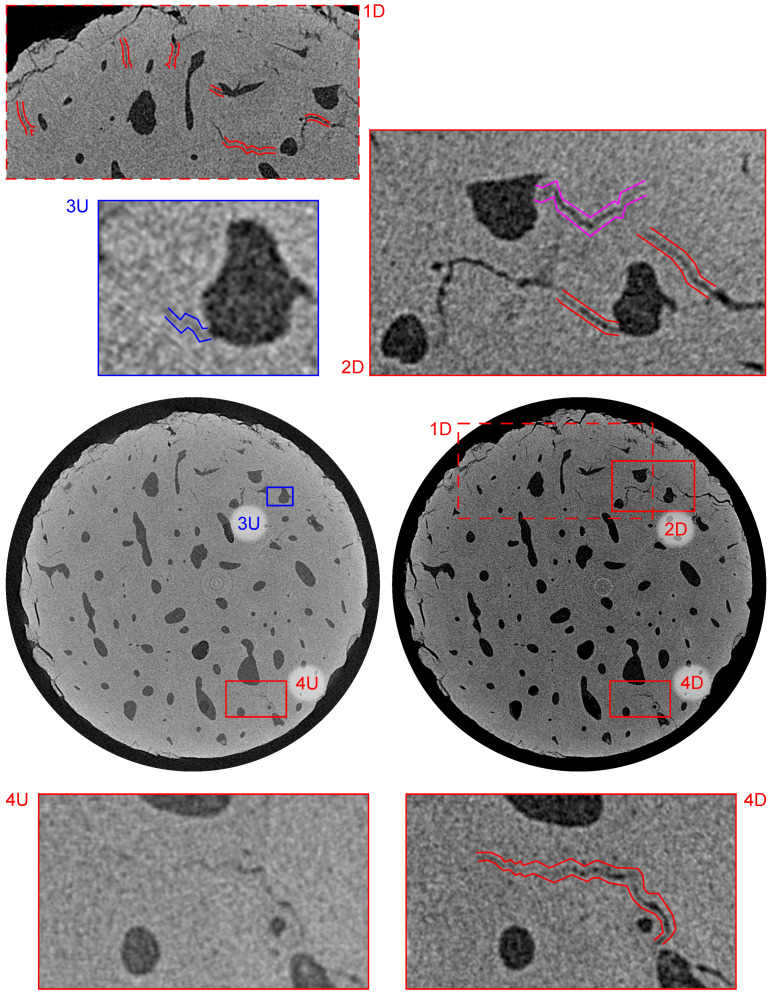
Visualization of the reconstructed 3D volumes—transverse plane at a distance of 2.64 mm from the upper loading platen. Index “U” stands for intact (unloaded) state and “D” stands for loaded (damaged) state. Blue color depicts small microcrack present in the intact state, purple color depicts microcrack formed by the fatigue loading, and red color depicts microcracks with increased dimensions as a result of the loading procedure.

**Figure 10:**
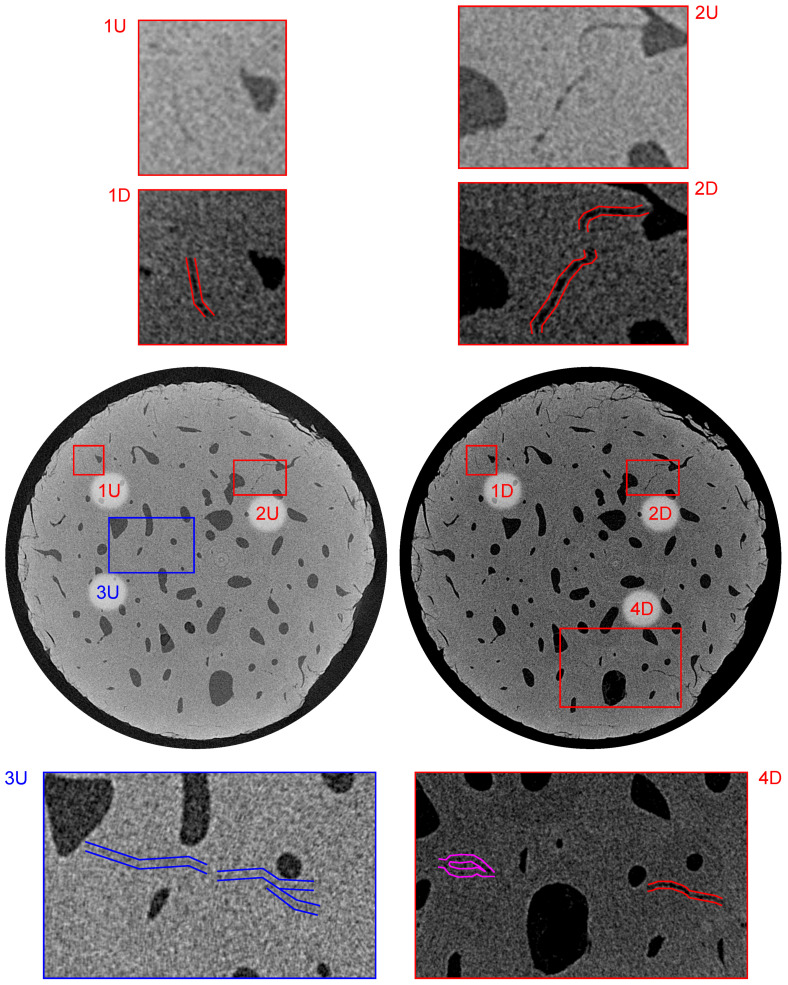
Visualization of the reconstructed 3D volumes—transverse plane at a distance of 2.02 mm from the upper loading platen. Index “U” stands for the intact (unloaded) state and “D” stands for the loaded (damaged) state. The blue color depicts the small microcrack present in the intact state, the purple color depicts the microcrack formed by the fatigue loading, and the red color depicts the microcracks with increased dimensions as a result of the loading procedure.

**Figure 11:**
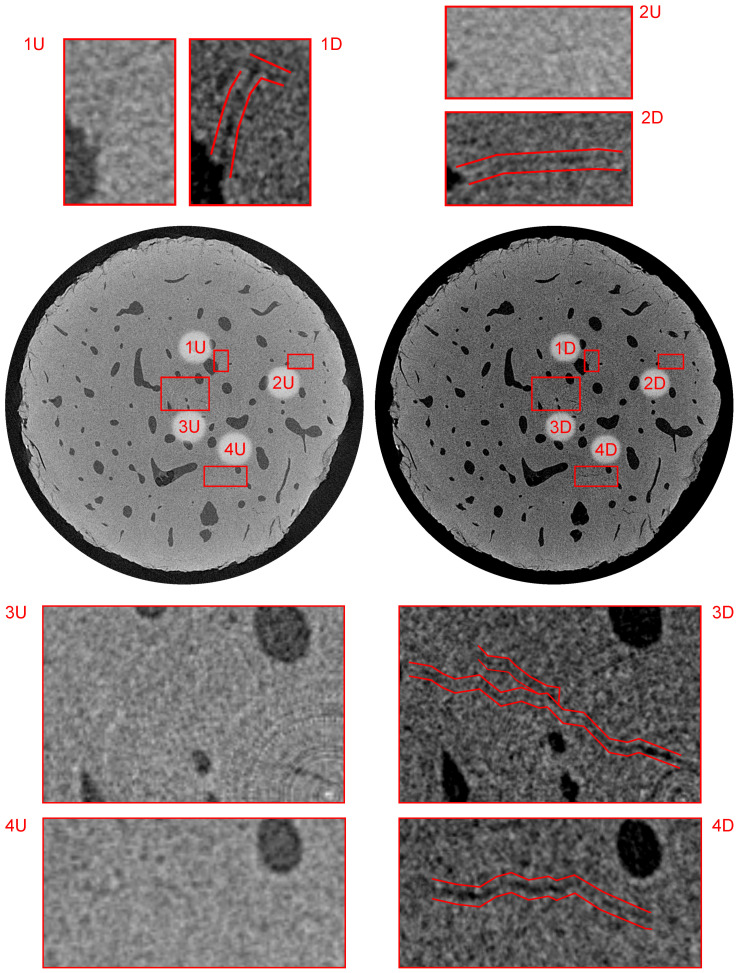
Visualization of the reconstructed 3D volumes—transverse plane at a distance of 1.76 mm from the upper loading platen. Index “U” stands for the intact (unloaded) state and “D” stands for the loaded (damaged) state. The figure depicts, in red color, the microcracks with increased dimensions as a result of the loading procedure.

The combination of the in-house loading instrumentation and CT imaging with a resolution of ≈2μm coupled with the post-processing based on the differential tomography methodology enabled us to reveal several microstructural features within the investigated bone sample. In the following paragraphs, we present the comparison between the 3D images of the intact sample (index “U” in Figure 9, Figure 10 and Figure 11) with the differential 3D image (index “D” in Figure 9, Figure 10 and Figure 11) at various locations in both the sagittal and transverse plane). In all the cases, the alignment of the volumes was performed using the transformation determined by the DVC, while only the microcracks not originating from the surface of the mechanically and thermally affected sample during the preparation are highlighted and taken into account.

Figure 9 shows the bottom part of the sample in the transverse plane at a distance of 2.64 mm from the upper loading platen. The red offset lines in the sub-figures 1D, 2D, and 4D show the system of microcracks in this slice that were also present in the intact sample and the fatigue loading procedure caused the increase of both the width up to 20μm and length including the interconnection of two previously isolated microcracks. The visualization of this process is provided in sub-figures 4U and 4D showing the typical behavior of such structural defects. Furthermore, this slice was also interesting for the occurrence of a very thin microcrack and a formation of a new microcrack. The very small microcrack identifiable in the 3D image of the intact sample is depicted in the sub-figure 3U and it shows that microcracks as narrow as 2 voxels (4μm) can be identified even if the sample is placed in the in situ loading device and the imaging parameters including the geometrical magnification are optimized for the time-lapse CT imaging. The purple offset lines in sub-figure 2D show a newly formed microcrack originating from the Haversian canal, which acts as a stress concentrator. Here, it can be seen that the presence of Haversian canals in the microstructure of the bone led to a reduction in the effective cross-section, which causes a stress redistribution around and between the canals and influences the crack formation during cyclic fatigue loading.

Figure 10 shows the bottom part of the sample in the transverse plane at a distance of 2.02 mm from the upper loading platen. The red offset lines in sub-figures 1D, 2D, and 4D again show the system of microcracks in this slice that were also present in the intact sample and the fatigue loading procedure caused the increase in the width in this case, where the width after loading was approximately 13μm (1D, 4D) and 22μm (2D). Sub-figures 1U/D and 2U/D document the process of the crack growth from the Haversian canals. Similar to Figure 9, sub-figure 3U depicts a visualization of a thin microcrack in the intact volume with its width at the resolution limit of the imaging setup. The measured length of this microcrack was 543μm. Furthermore, the purple offset lines in sub-figure 4D show a newly formed microcrack located in this slice between three Haversian canals.

Figure 11 shows the bottom part of the sample in the transverse plane at a distance of 1.76 mm from the upper loading platen. This figure demonstrates the process of microcrack identification in the unloaded state at the limit width of two voxels (4μm) and the growth of the microcracks during fatigue loading resulting in the increase of both width and length. However, the most important information presented in this image is the ability to perform such an identification near the axis of rotation. Here, the presence of ring artifact arising from imaging instrumentation characteristics causes problems in the identification of morphological features and reliability of mathematical operations over reconstructed 3D images. It can be seen in sub-figures 3U/D that the instrumentation and post-processing methods presented in this paper enable to identify the microcrack at the resolution limit of the experiment almost directly at the rotation center of the specimen and after the blending procedure.

**Figure 12:**
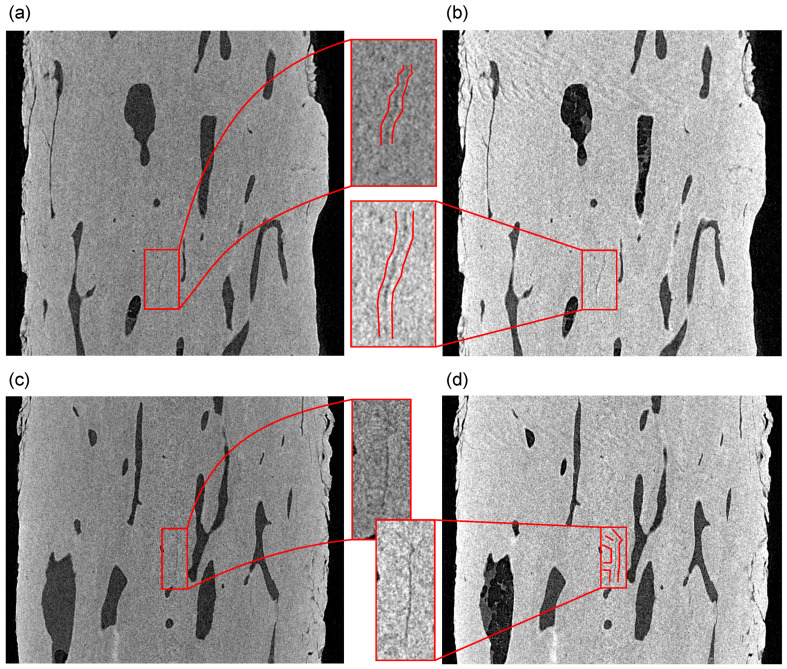
Perpendicular slices through the reconstructed 3D volumes at the symmetry axis of the specimen: (**a**,**b**) sagittal and (**c**,**d**) coronal plane. The growth of the microcracks is shown in comparison to the intact state (**a**,**c**) and the loaded state (**b**,**d**). The figure depicts in red color the microcracks with increased dimensions as a result of the loading procedure.

Moreover, the negative effects of the ring artifact on the rotation axis may be more pronounced in the case of any microcracks oriented vertically. Figure 12 depicts such a situation on the two perpendicular visualizations of the specimen in the sagittal and coronal plane at the symmetry axis of the specimen. It can be seen in both slices that the microcracks are identifiable near the rotation axis in both the intact state and after the fatigue loading of the specimen, including its branching towards the rotation axis.

**Figure 13:**
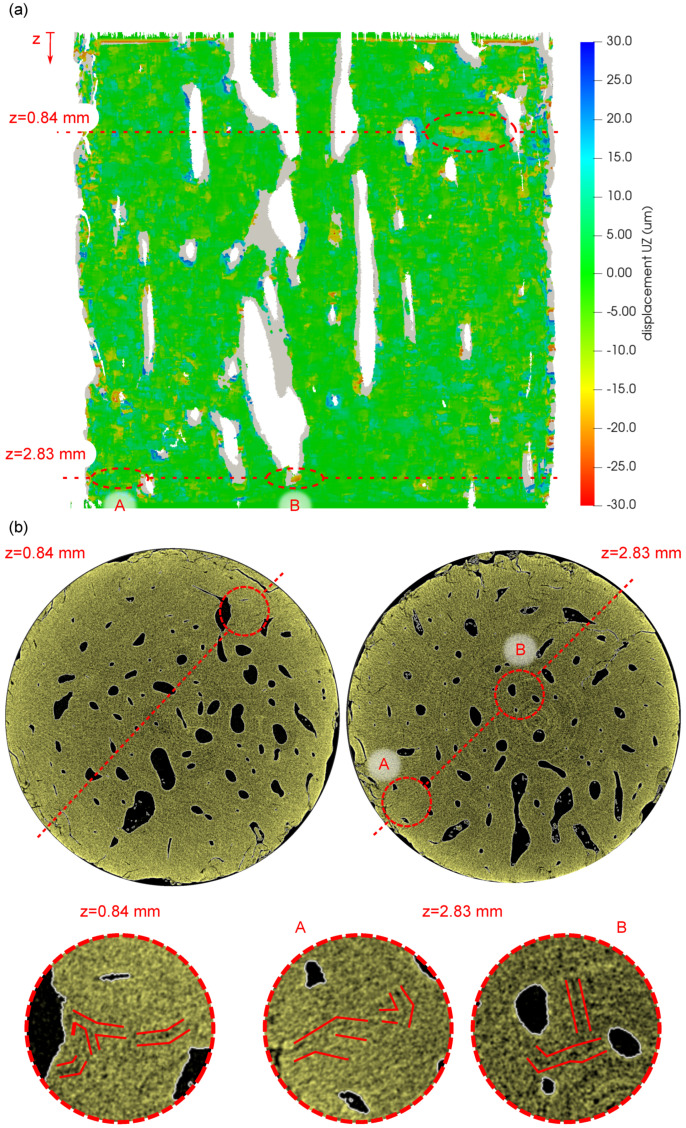
The results of the DVC procedure and comparison with the transversal slices in the blended volume: (**a**) $u_{z}$—vertical displacement field calculated in “plane 1”, (**b**) slices and details of the bone microstructure at the coordinates z=0.84mm and z=2.83mm, where regions **A** and **B** show detailed views on the identified microcracks).

Figure 13 shows the results of the DVC procedure performed on two perpendicular planes intersecting several Haversian canals of the investigated bone sample and the relationship of the calculated vertical displacements ($u_{z}$) to the system of fatigue-induced microcracks. In Figure 13a, the visualization of the $u_{z}$ displacement field is presented together with the position of two slices at coordinates z=0.84mm and z=2.83mm selected for comparison with the visualization of the blended volume. In Figure 13a, the grey areas depict the regions with the lost correlation, while the highlighted regions with either the high compressive displacement or the regions near the Haversian canals were selected as probable locations with expected fracture damage for further inspection in the blended volume. Figure 13b depicts the visualizations of the selected slices in the blended volume showing both the whole cross-section of the sample and details of the regions of interest corresponding to the areas identified in the displacement field. It can be seen that in every case that the selected region contains a system of microcracks, which demonstrates the applicability of the full-affine DVC procedure for the identification of the fatigue damage in such a bone sample subjected to the gait cycle-derived loading.

## 4. Discussion

The high resolution time-lapse XCT under loading was carried out using in-house instrumentation based on a laboratory X-ray scanner to investigate the damage evolution in a human bone sample subjected to low cycle fatigue simulating a typical gait cycle. The reconstructed 3D images were subjected to two different formulations of the DVC procedure to enable the precise alignment of volumes for the differential CT evaluation procedure and to calculate the displacement field in the selected medial planes of the sample. On the basis of the acquired results, the following comments, findings, and remarks can be drawn:

- It was possible to identify fatigue microcracks and their propagation in the entire volume of the investigated sample using the laboratory X-ray scanner and custom post-processing procedures. The ability to identify the system of microcracks is essential to understand the bone failure mechanisms on the microscopic level, particularly regarding the fact that the microscopic damage of the bone and the rate of its accumulation plays a significant role in the reduction of the bone bearing capacity in the post-yield regime. The fully volumetric analysis, in this case, also brings advantages over an investigation based on 2D slices, both in terms of the microcrack identification and quantification, which is given by their complex arbitrary shape in the microstructure of the bone.
- In all the cases, it was found out, at the achieved voxel size, that the existing or newly formed microcracks either originate from the stress concentrators in the microstructure of the bone or interconnect them. In the investigated samples, the stress concentrators can be divided into three groups comprising the microcracks present in the intact state, the cracks on the surface of the specimen created during the manufacturing of the sample, and the Haversian canals. Although this is an expected result conforming to the fundamentals of fracture mechanics, it is an important finding that the regions of stress concentration with the presence of a system of microcracks were clearly apparent in the displacement field of the full-affine DVC procedure. With respect to the 2 micron resolution achieved in the reconstructed 3D images of the bone, the statistics from the two perpendicular planes, where the full-affine DVC was evaluated, shows approximately a 50% probability that a microcrack is present at the strain concentration location. This is still, however, a valuable result since the automated identification of the microcracks can be limited only to the regions of interest determined from the DVC with implications on computational costs and a time reduction. Conversely, no microcracks in the regions of the uniform strain distribution were identified at this scale level limited by the micrometric voxel size.
- It is possible to perform the in situ fatigue loading of a cylindrical human cortical bone sample using a laboratory CT scanner at a resolution given by the geometry of the loading device influencing the achievable source-to-object distance, and thus the achievable geometrical magnification of the projections. This enabled us to identify the microcracks in both the intact sample and after the loading procedure, with the width of at least 2 voxels, i.e., ≈4μm. Due to the nature of the CT imaging, regarding the conclusions, the probable presence of microcracks with dimensions under the resolution limit of the imaging instrumentation is questionable. However, this factor does not necessarily decrease the impact of this study as the aim was to show the methodology based upon a laboratory CT scanner, where the DVC using full-affine transformation serves as a tool for the identification of the damage accumulation in the bone represented by a system of microcracks.
- However, it is necessary to take appropriate measures with laboratory CT scanners to achieve the sufficient quality of the resulting reconstructed 3D images. Thus, the achieved value of the geometrical magnification of the projections is only one of the parameters determining the overall quality of the radiographical imaging. To reduce the influence of various tomographical artifacts including ring artifacts and the beam hardening effect, appropriate corrections of the imaging detector and X-ray source characteristics have to be performed to sharpen the reconstructed 3D image and enable the reliable identification of the microscopic features in the bone. It can be seen in the projections that the beam hardening effect consists of a higher intensity in the vicinity of the surface as no particular treatment was applied to its reduction. However, since the evaluation near the surface of the sample was omitted due to the damage induced by the sample preparation, the beam hardening itself has only a negligible influence on the acquired results. Furthermore, we have shown in the FWHM comparison with the SEM imaging that a combination of projection-level corrections with the focal spot drift correction leads to a quality of the reconstructed 3D images comparable to the SE microscopy in terms of the crack thickness calculation and void-material interface identification.
- Since the aim of the study was to show the methodology combining in situ fatigue loading using a simulated gait cycle with a DVC based evaluation and differential tomography for the identification of the microcracks present in the microstructure of the bone, only one sample was subjected to the full evaluation procedure comprised of the discussed methods. Nevertheless, in total, six samples were tested using the in situ loading procedure, but the acquired results were unsatisfactory due to several reasons. During two tests, problems were encountered with the mechanical response of the sample resulting in its sudden disintegration during the loading procedure, presumably due to the thermal and mechanical damage inflicted during the sample preparation. Generally, due to the variability in the mechanical response of the samples, it was difficult to determine the number of required cycles to generate microcracks, but prevent the destruction of the sample. Additionally, the behavior of the microstructure may also result in the closing of the microcracks, which corresponds to the behavior of the bone in the human body, but such a process precludes a study of the crack formation using time-lapse radiographical imaging. Additionally, the long-term stability of the detector used for imaging has an influence on the noise in the reconstructed 3D images and their sharpness, where the achieved geometrical resolution with a reduced reconstruction quality may also prevent the evaluation of the experiment.

## 5. Conclusions

The possibility to investigate the system of fatigue microcracks in a human cortical bone using laboratory X-ray imaging instrumentation was studied. A combination of an in-house developed X-ray CT scanner and a loading device suitable for the in situ time-lapse radiographical imaging of biological samples under loading was used in the experiments. The investigated cylindrical bone sample was subjected to low-cycle fatigue loading using a simulated gait cycle directly in the CT scanner, resulting in the damage development within the microstructure of the bone. Using the reconstructed 3D images of the bone in the intact state and after the loading procedure, the developing system of microcracks was studied using the in-house DVC procedure and a differential tomography approach. It has been shown that the utilized instrumentation and evaluation procedures can be successfully used for the identification of microcracks with a width of at least 4μm, while the DVC can be used as a tool for the inspection of the deforming microstructure of the bone for damage accumulation regions, which were clearly apparent in the calculated displacement fields. It has been found out that a microcrack or system of microcracks is present in the displacement concentration indicated by the DVC with approximately 50% probability, which still enables significant computational cost reduction during automated evaluation of damage. Even though the results have to be evaluated with respect to the achieved voxel size, the proposed method shows that laboratory-based instrumentation can be used for damage assessment at this scale level, which is an important achievement in the field of tissue engineering including bone scaffold development. In further studies, the work will be concentrated on the resolution increase and improvement of artifact reduction methods without negative effects on the sharpness of the reconstructed 3D images. Simultaneously, it is necessary to enhance the experimental methods to increase the statistical impact of the results.

## Figures and Tables

**Table 1:** The results of the determination of the optimal correlation window size. NaN indicates the number of nodes, where the unconverged solution occurred, the value of the standard deviation $\sigma_{\mathrm{YZ}}$ shows the error from the ideal displacement $u_{\mathrm{YZ}}$ given by the length of the motion vector ($\sqrt{20^{2}+20^{2}}=28.2842$) in the YZ plane.

Window Size	Elapsed Time	NaN	Mean $u_{\mathrm{YZ}}$	$\sigma_{\mathrm{YZ}}$	MBE	RMSE
ine [px]	[s]	[nodes]	[px]	[px]	[-]	[-]
ine 6	2674	159	24.38	3.9	3.9	6.73
8	2521	83	27.13	1.15	1.15	3.34
10	2627	47	27.92	0.37	0.37	1.72
12	3161	1	28.25	0.04	0.04	0.43
14	6915	0	28.28	$4.4\times 10^{-3}$	$4.4\times 10^{-3}$	0.15
16	6028	0	28.28	$7.15\times 10^{-5}$	$1.02\times 10^{-4}$	$1.27\times 10^{-4}$
18	3423	0	28.28	$1.0\times 10^{-4}$	$1.4\times 10^{-4}$	$1.65\times 10^{-4}$
20	4117	0	28.28	$7.13\times 10^{-5}$	$1.13\times 10^{-4}$	$1.38\times 10^{-4}$

## Abbreviations

The following abbreviations are used in this manuscript:

API	Application Programming Interface
CMOS	Complementary Metal Oxide Semiconductor
CT	Computed Tomography
DIC	Digital Image Correlation
DVC	Digital Volume Correlation
FBP	Filtered Back-Projection
FDK	Feldkamp, Davis, and Kress (CT reconstruction algorithm)
GOS	Gadolinium oxysulfide
FWHM	Full-Width Half-Maximum
MBE	Mean-Bias Error
NCC	Normalized Cross-Correlation
RMSE	Root-Mean Square Error
SEM	Scanning Electron Microscopy
VOI	Volume Of Interest
XCT	X-ray (micro) Computed Tomography
